# Supplementary figures and images for: Candidate Transcript Panel in Semen Extracellular Vesicles Can Improve Prediction of Aggressiveness of Prostate Cancer
Source: Int J Mol Sci. 2025 Sep 30;26(19):9562. doi: 10.3390/ijms26199562 (PMC12525505; doi:10.3390/ijms26199562)

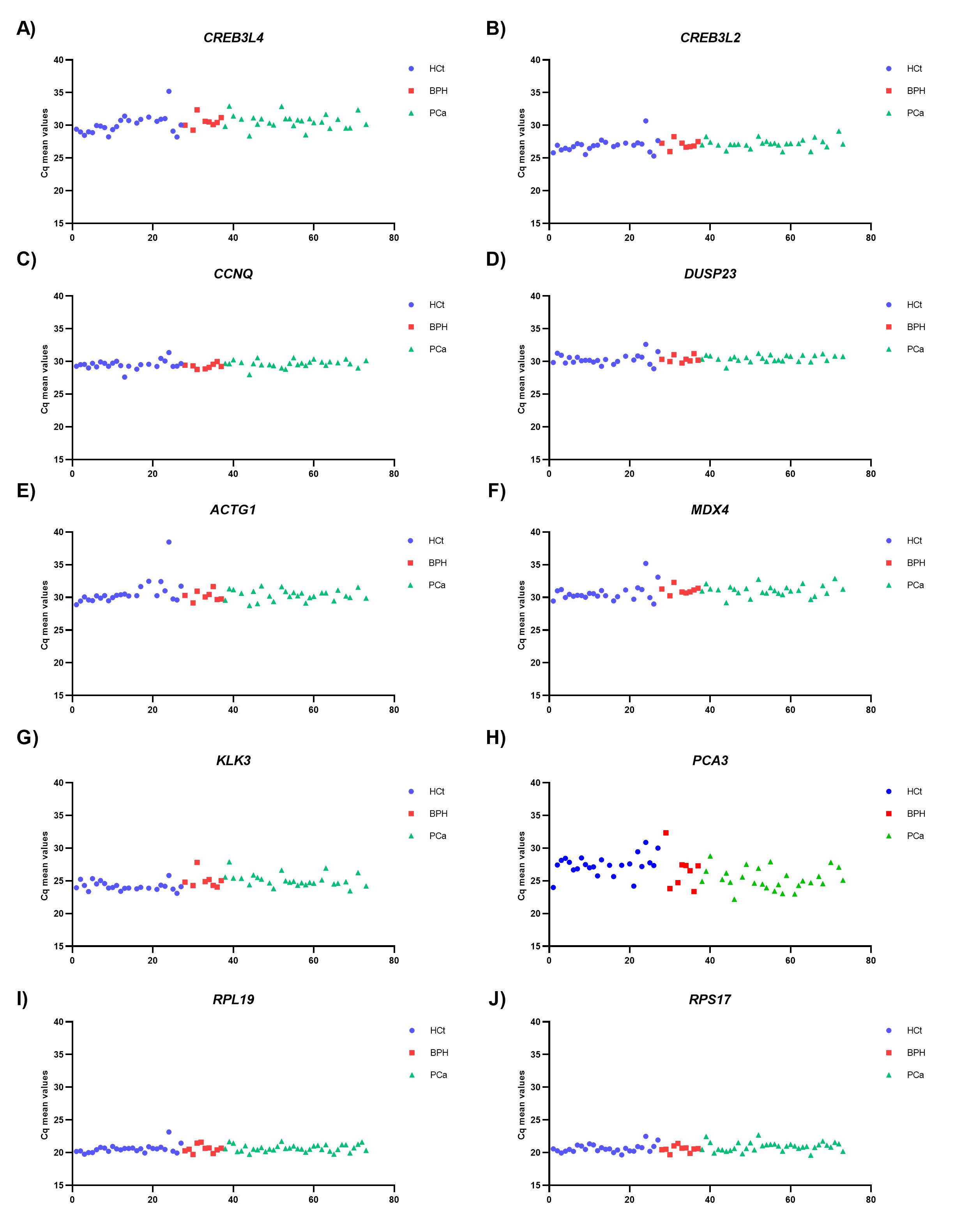

Supplement: Supplementary file 1 [file ijms-26-09562-s001.zip › Suppl Figure S1_new.jpg]

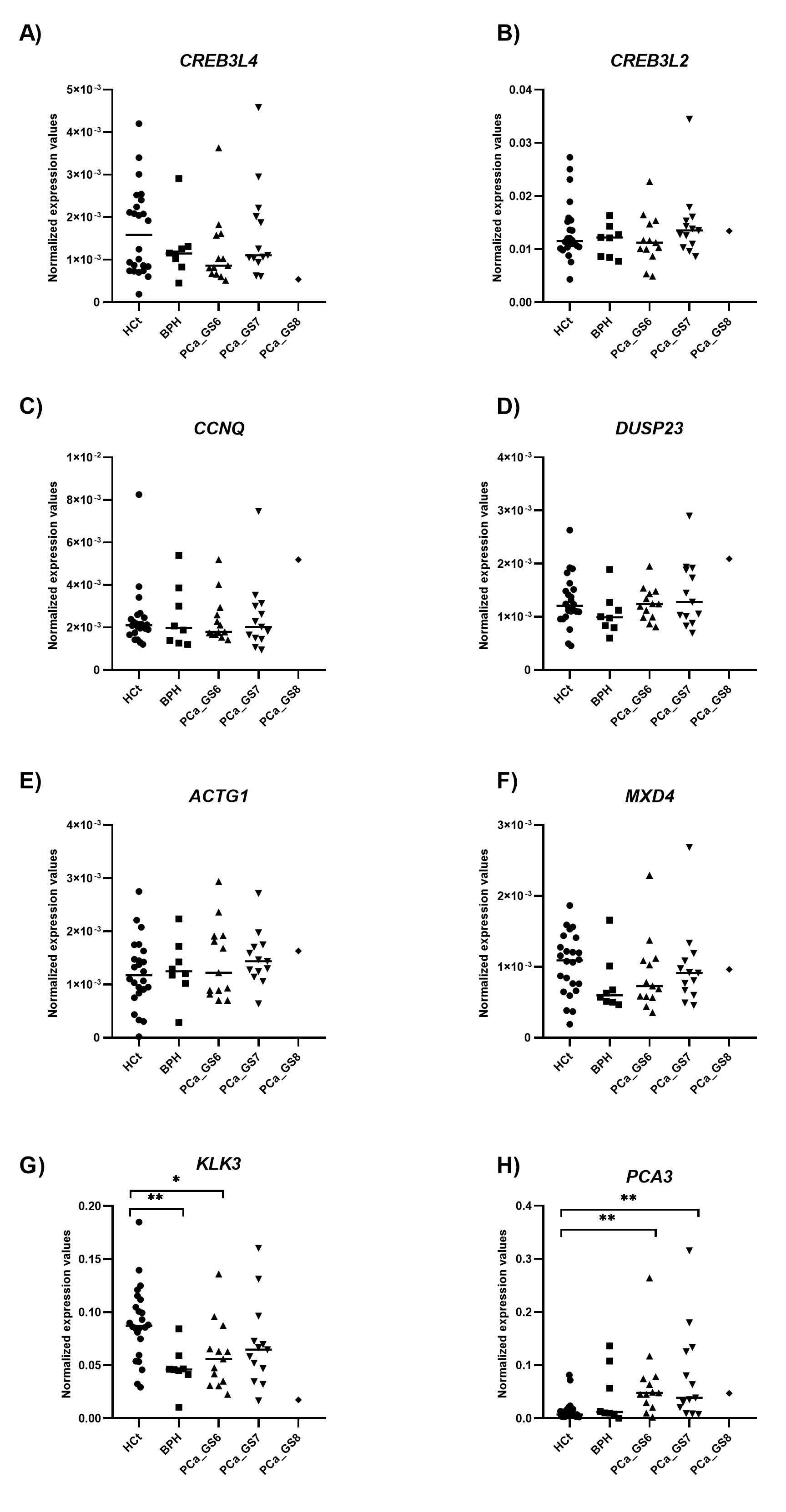

Supplement: Supplementary file 1 [file ijms-26-09562-s001.zip › Suppl Figure S2_prev Suppl Figure S1.jpg]

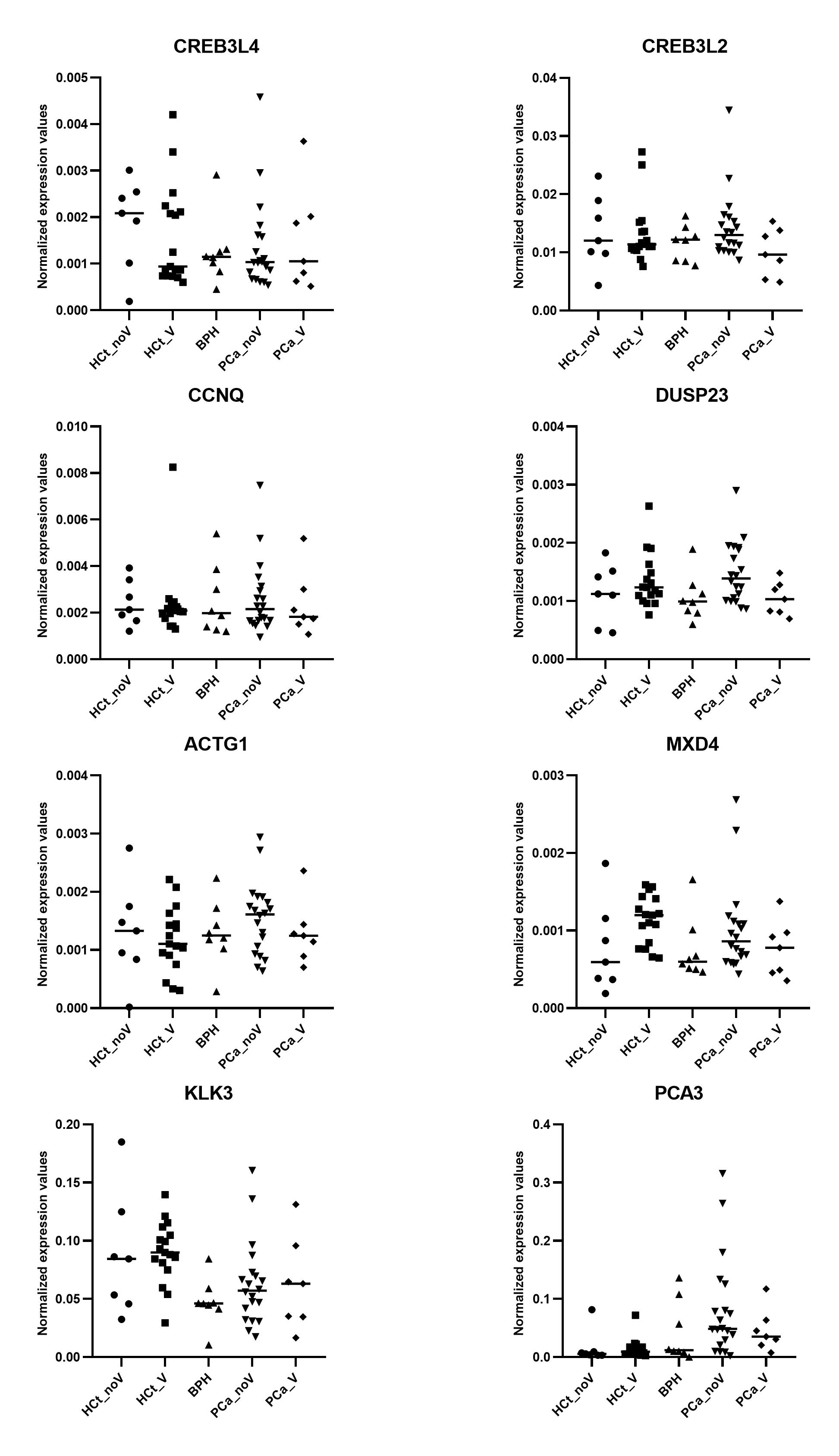

Supplement: Supplementary file 1 [file ijms-26-09562-s001.zip › Suppl Figure S3_prev Suppl Figure S2_Fenotipo_noV_V_no SERINC4.jpg]
